# WHITE MATTER CHANGE NEAR CEREBRAL MICROBLEEDS AFTER MTBI INVOLVES AGE AND SEX DEPENDENT COGNITIVE DECLINE

**DOI:** 10.1093/geroni/igac059.2835

**Published:** 2022-12-20

**Authors:** Andrei Irimia, Van Ngo, Nikhil Chaudhari, Fan Zhang, Shantanu Joshi, Lauren O’Donnell, Nasim Sheikh-Bahaei, Helena Chui

**Affiliations:** University of Southern California, Los Angeles, California, United States; University of Southern California, Los Angeles, California, United States; University of Southern California, Los Angeles, California, United States; Harvard Medical School, Boston, Massachusetts, United States; University of California, Los Angeles, Los Angeles, California, United States; Harvard Medical School, Boston, Massachusetts, United States; University of Southern California, Los Angeles, California, United States; University of Southern California, Los Angeles, California, United States

## Abstract

We tested the null hypothesis that, after mild traumatic brain injury (mTBI), white matter changes near cerebral microbleeds (CMBs) are associated with cognitive decline. Magnetic resonance images were acquired from 62 adults with mTBI and from 203 matched healthy controls. A week post-injury, mTBI participants had 2.7±2.6 traumatic CMBs in WM, located 6.1±4.4 mm from the cortical mantle. About 6 months later, 97% of CMBs were associated with significant reductions (34%±11%, q < 0.05) in the fractional anisotropy (FA) of WM streamlines within ~1 cm of CMBs. Male sex and older age were significant risk factors for larger reductions (q < 0.05). CMBs in the corpus callosum, cingulum bundle, inferior and middle longitudinal fasciculi were associated with FA changes that were significantly and positively associated with changes in cognitive functions mediated by these structures (q < 0.05). These findings distinguish non-traumatic from traumatic CMBs according to CMB-related changes in surrounding WM. Our findings also challenge the assumption that traumatic CMBs are cognitively silent and identify older age and male sex as risk factors for mTBI-related cognitive decline in the presence of CMBs. In conclusion, mTBI with CMB findings on MRI can be described as a clinical endophenotype that warrants longitudinal mapping and quantification of cognitive function.

